# Metabolic Consequences of Anabolic Steroids, Insulin, and Growth Hormone Abuse in Recreational Bodybuilders: Implications for the World Anti-Doping Agency Passport

**DOI:** 10.1186/s40798-024-00697-6

**Published:** 2024-03-27

**Authors:** Filippo Giorgio Di Girolamo, Chiara Biasinutto, Alessandro Mangogna, Nicola Fiotti, Pierandrea Vinci, Rado Pisot, Filippo Mearelli, Bostjan Simunic, Chiara Roni, Gianni Biolo

**Affiliations:** 1 Department of Medical, Surgical and Health Sciences, University of Trieste, Trieste, Italy; 2 University of Primorska, Koper, Slovenia; 3 Science and Research Centre Koper, Institute for Kinesiology Research, Koper, Slovenia; 4 SC Assistenza Farmaceutica e Territoriale, Azienda Sanitaria Universitaria Giuliano Isontina, – Trieste, Trieste, Italy; 5 UCO Clinica Medica, Azienda Sanitaria Universitaria Giuliano Isontina, Trieste, Italy; 6 Institute for Maternal and Child Health, Istituto di Ricovero e Cura a Carattere Scientifico (IRCCS) Burlo Garofolo, Trieste, Italy; 7 Ospedale di Cattinara, SC Assistenza Farmaceutica e Territoriale, Strada di Fiume, 447, Trieste, 34149 Italy

**Keywords:** Doping, Drug abuse, Bodybuilders, Metabolism

## Abstract

**Background:**

Hormonal doping in recreational sports is a public-health concern. The World Anti-Doping Agency (WADA) promoted the creation of the Athlete Biological Passport, aiming to monitor athlete’s biological variables over time to facilitate indirect detection of doping. Detection tests for anabolic androgenic steroids (AAS) and growth hormone (GH) are available while insulin abuse cannot be revealed. We have determined in recreational bodybuilders the metabolic effects associated with different patterns of hormone abuse. All analyses were conducted using Statistical Package for Social Sciences (SPSS) 21.0 software (SPSS Chicago, IL).

**Results:**

We have assessed plasma concentrations of selected metabolic markers and fatty acid content in erythrocyte membranes of 92 male bodybuilders and in 45 healthy controls. Hormonal abuse was identified by anonymous questionnaires. 43% (%) of recruited bodybuilders regularly abused hormones, i.e., anabolic androgenic steroids (95%) often associated with GH (30%) and/or insulin (38%). HDL-cholesterol was lower in insulin and/or GH abusers. Alanine (ALT) and aspartic (AST) transaminases were greater in hormone abusing bodybuilders than in non-doping bodybuilders and controls. Insulin doping was selectively associated with increased plasma ALT-to-AST ratio. In erythrocyte membranes, elongase activity (i.e., stearic-to-palmitic ratio) was lower in insulin and/or growth hormone doping, whereas increased Δ-9 desaturase activity (i.e., palmitoleic-to-palmitic ratio) was selectively associated with insulin doping.

**Conclusions:**

In conclusion, our study demonstrates that insulin and GH abuse are characterized by multiple alterations of specific metabolic markers. Although further studies are needed to test whether longitudinal monitoring of selected metabolic marker such as muscle contraction time, HDL levels, ALT-AST ratio as well as the activities of selected enzymes (e.g. Δ-9 desaturase and elongase), could contribute to the detection of insulin and GH abuse in sport.

**Supplementary Information:**

The online version contains supplementary material available at 10.1186/s40798-024-00697-6.

## Key Points

1. Drug abuse is common among bodybuilder athletes. Substances combinations can result in significant health risks, including cardiovascular events, hormonal imbalances, and metabolic complications.
2. The World Anti-Doping Agency promoted the creation of the Athlete Biological Passport, aiming to monitor athlete’s biological variables over time to facilitate indirect detection of doping. Detection tests for anabolic androgenic steroids (AAS) and growth hormone (GH) are available while insulin abuse cannot be revealed.
3. The present study investigates the effects of insulin, GH, and AAS abuse on selected metabolic parameters of recreational bodybuilders to identify selective, sensitive markers useful in longitudinal doping detection and, possibly, to be incorporated in the WADA Athlete Biological Passport.

**Supplementary Information:**

The online version contains supplementary material available at 10.1186/s40798-024-00697-6.

## Background

Drug abuse, with the aim of improving muscle mass and physical performance, is frequently observed among bodybuilder athletes, being anabolic androgenic steroids (AAS), growth hormone (GH), and insulin being some of the most commonly used substances [1].

The anabolic properties of AAS have been widely established in humans [2]. Supraphysiological levels of testosterone have positive anabolic effects on the musculoskeletal system, influencing lean body mass, muscle size, and protein metabolism [3–6] in hypogonadal men, as well as in healthy young and elderly individuals [7–9]. Moreover, a growing body of evidence suggests that AAS improves muscle strength, collagen synthesis, and positively impacts bone metabolism [10–13].

In addition to steroid hormones, naturally released peptide and protein hormones, such as GH and insulin-like growth factor-1 (IGF-1), appear to increase the following resistance training (RT) [14, 15]. Some studies have shown a potential connection between the rise in GH after RT and long-term muscle hypertrophy [15], while others have not consistently supported these findings [16, 17]. GH leads to an increase in serum IGF-1 levels [18], which may indicate anabolic effects [1]. Regardless of the precise pharmacological mechanisms, GH became popular as a performance-enhancing drug in the early 1990s, especially with the availability of its recombinant form [19]. Tracer studies employing a steady-state technique have provided valuable insights into the regulatory role of GH in the whole-body protein anabolism. Indeed, GH plays a pivotal role in redirecting amino acids away from oxidative pathways and towards synthetic pathways, thus promoting anabolism [20]. Additionally, IGF-I mirrors the effects of GH by stimulating protein synthesis and concurrently reducing oxidation. This observation strongly supports the notion that IGF-I plays a crucial role in mediating the impact of GH on the overall protein anabolism of the entire body [21]. GH abuse also relates to its ability to induce lipolysis during both periods of rest and exercise, resulting in increased plasma fatty acid levels and consequent fat oxidation. Additionally, GH raises plasma glucose levels by enhancing glycogenolysis and gluconeogenesis [22–26]. Consequently, GH has the potential to augment muscle function by bolstering the availability of fatty acids and pyruvate as metabolic substrates for energy generation. Together, exercise prompts an elevation in cardiac output and directs increased blood flow to the muscles engaged in physical activity [22–26]. The significant enhancement in local perfusion efficiently channels substrates to their most crucial destinations, aiding in the removal of lactate from active muscles and transporting it to the liver for recycling into glucose through the Cori cycle [22–26]. These concepts could explain the widespread abuse of GH in sport and its perception as a potent anabolic drug and the resulting challenges associated with detection. However, studies have shown that, when administered under controlled supervision at regulated doses, GH doesn’t have an impact on strength or endurance but only demonstrates selective improvement in sprinting ability [21].

Insulin is frequently used among bodybuilders and strength athletes [27], even without direct evidence of its anabolic effects [1]. An increasing number of athletes without diabetes voluntarily use insulin, and evidence suggests that the extent of misuse is considerable and growing [28]. Insulin enhances glucose uptake and maximizes glycogen storage before exercise, potentially improving performance [29]. Muscle glycogen stores are the primary carbohydrate source during exercise, and their content determines the maximal exercise duration. Insulin is responsible for the shift from muscle catabolism associated with overnight fasting to the anabolic response to feeding.

Numerous investigations have displayed the potential of insulin to augment amino acid transport, thereby indirectly facilitating the synthesis of contractile proteins while impeding their degradation [27, 30]. Furthermore, there is evidence indicating that the anabolic response of insulin is raised in conjunction with protein or amino acid intake [31–34]. Following intravenous administration of insulin, there is a dose-dependent reduction in circulating plasma amino acid levels, with branched-chain amino acids demonstrating heightened sensitivity to increased insulin levels [34]. This insulin-induced hypoaminoacidemia signifies an increased uptake of amino acids from the plasma, accompanied by the suggested inhibitory influence of elevated insulin on endogenous proteolysis [34]. The underlying hypothesis proposes that the positive impact of exogenous insulin on muscle protein synthesis stems from insulin-induced enhanced blood flow, leading to a greater delivery of amino acids to the muscle. Nonetheless, a decline in circulating amino acid concentrations may limit the delivery of amino acids to the muscle, potentially hindering the subsequent upsurge in muscle protein synthesis.

Nevertheless, the lack of an appropriate control group exhibiting a similar degree of hyperaminoacidemia presents a significant challenge in distinguishing between the purported anabolic effects of insulin and amino acid administration in these studies. Several studies investigating whether exogenous insulin administration can further amplify muscle protein synthesis during hyperaminoacidemic conditions consistently failed to discern an incremental effect [34]. These outcomes suggest that while concurrent hyperinsulinemia and hyperaminoacidemia may elevate muscle protein synthesis, particularly in healthy young subjects, this effect seems predominantly attributed to hyperaminoacidemia. In a study employing a clamp-based approach, exogenous insulin was administered to attain local supraphysiological insulin levels surpassing 50,000 pmol/l, while amino acids were clamped at basal arterial or venous levels [35]. This study reported elevated rates of muscle protein synthesis, indicating that supraphysiological insulin levels may indeed effectively stimulate muscle protein synthesis. However, it is conceivable that self-administration of short-acting insulin, in tandem with carbohydrate intake in a healthy individual, may blunt endogenous insulin production without a substantial elevation in net insulinemia. Similarly, the principle applies to long-acting insulin, which predominantly suppresses beta cell function without exerting a significant impact on circulating insulin levels [36].

Improper use of insulin can result in severe hypoglycemia [37] and increased fat accumulation, elevating the risk of obesity and related diseases [38]. The technique of abuse is relatively simple: users self-inject short-acting insulin subcutaneously and consume sugar-containing foods and/or drinks to prevent hypoglycemic events before or after workouts [1].

The abuses of GH, insulin, and AAS are often combined to potentially achieve additive or synergistic effects [39]. The simultaneous use of insulin and AAS abuse is widespread among bodybuilders and athletes, with 21% of male bodybuilders admitting to using steroids, and approximately 7% of them concurrently using insulin [28], adding to the prevalence of athletes using AAS, GH, and insulin in combination [10].

The illicit use of these drugs in non-competitive bodybuilders can result in significant side effects. Androgen use can adversely affect lipid profiles by increasing LDL cholesterol and decreasing HDL cholesterol levels [40]. It can also induce hepatotoxicity, leading to elevated liver enzyme levels and liver damage [41], and suppress natural testosterone production, resulting in hypogonadotropic hypogonadism, i.e. testicular atrophy, reduced fertility, and decreased sperm production [42]. Supraphysiological doses of growth hormone can lead to several metabolic complications, such as insulin resistance and an increased risk of type 2 diabetes [43]. Combining androgens, growth hormone, and insulin in non-competitive athletes can result in complex interactions and potentially increase the risk of cardiovascular events (myocardial infarction, and stroke) [44, 45] and further disrupt endocrine function i.e., the hypothalamic-pituitary-gonadal axis with infertility, sexual dysfunction, and alterations in mood and behavior [46].

In its fight against doping, The World Anti-Doping Agency (WADA) promoted the creation of the Athlete Biological Passport, whose fundamental principle is based on “the monitoring of an athlete’s biological variables over time to facilitate indirect detection of doping on a longitudinal basis, rather than on the traditional direct detection of doping.” Detection tests for AAS and GH are available [16, 39], whereas insulin abuse cannot be revealed by traditional laboratory techniques [39]. Insulin is produced by recombinant DNA techniques (biosynthetic human insulin). While mass spectrometric procedures are available in identifying degradation products of insulin analogs in human urine [47–49], recombinant human insulin cannot be detected by current methods because it is indistinguishable from naturally occurring insulin. In addition, circulating insulin exhibits a half-life of 5–10 min.

In the present study, we investigate the effects of insulin, GH, and AAS abuse on selected metabolic parameters in recreational bodybuilders to identify selective, sensitive markers useful in longitudinal doping detection and, possibly, considered for inclusion in the new WADA guidelines concerning the Athlete Biological Passport.

## Methods

### Study Design

The study followed an observational cross-sectional protocol. We recruited 92 recreational male bodybuilders and 45 healthy active male controls (total population *n* = 137) through advertisements in Slovenian recreational gyms and sports facilities. Inclusion criteria consisted of individuals aged > 18 and < 50 years, engaged in regular strength training for at least 2 years (4–5 sessions/week, 1–2 h/session), and a willingness to anonymously report illicit substance abuse. Exclusion criteria included participation in competitive sports, acute or chronic illnesses, and therapeutic use of these substances. A physician conducted a medical history and physical examination to exclude participants with chronic or acute illnesses, pharmacological treatment, and current smoking. All enrolled volunteers met the study criteria. Bodybuilders completed anonymous questionnaires to assess their habits related to illicit substance abuse, which were then matched with corresponding blood samples and body composition data. All bodybuilder volunteers were evaluated during the training periods preceding a contest. The study received approval from the National Medical Ethics Committee of the Republic of Slovenia (No. KME 21k/11/07), in accordance with the Declaration of Helsinki and its amendments. All volunteers provided written informed consent.

### Anthropometry and Body Composition

Standard methods were employed to measure anthropometric data, including body mass, height, waist, and hip circumference. Participants were weighed on an electronic scale while wearing only underwear after emptying their bladder. Body height was measured without shoes using a stadiometer. Body composition indices, specifically fat-free mass (FFM) and fat mass (FM), were determined using multifrequency bioimpedance (BIA-Human Implus–DSmedica, Milan, Italy). Bioelectrical impedance measurements were taken in the morning after an overnight fast, with participants lying in bed for 30 min before assessment to ensure body fluid redistribution. During this time, tensiomyographic assessment was performed. Bodybuilders were instructed to refrain from strenuous exercise starting the night before and to empty their bladder before examination. Measurements were conducted in accordance with the manufacturer’s instructions. Software provided by the manufacturer was used to calculate FM and FFM. The FFM index (FFMi) was computed as FFM (kg) divided by the square of height in meters (m²). None of the participants reported recent medication use that might affect body water compartments.

### Tensiomyography

Tensiomyography (TMG) was conducted on the vastus lateralis (VL) and biceps femoris (BF) muscles during electrically-evoked maximal isometric contractions. A single 1-ms maximal monophasic electrical impulse elicited a twitch contraction that caused muscle oscillations. These oscillations were recorded using a sensitive digital displacement sensor (TMG-BMC Ltd., Ljubljana, Slovenia) placed on the skin at the muscle measurement site. Stimulation amplitude was gradually increased until the twitch displacement amplitude (Dm in mm) reached its maximum, ranging from 85 to 110 milliamperes at a constant 30 volts. Contraction time (Tc in ms) was calculated from two maximal twitch responses as the time for amplitude to increase from 10 to 90% of Dm [49, 50].

BF measurements were performed with participants in a prone position, with a knee angle set at 5° flexion using foam pads. The measuring site for BF was halfway between the ischial tuberosity and the posterior knee joint fold, along the line of the BF long head. VL measurements were conducted with participants in a supine position, with a knee angle set at 30° flexion, also using foam pads. The measuring point for VL was located at 30% of the distance between the knee joint and the anterior superior iliac spine.

### Biochemistry

Blood samples (20 mL) were collected from the forearm vein of all participants in the morning after an overnight fast. After centrifugation at 3000 g at 4 °C for 10 min, plasma and erythrocytes were processed according to analytical protocols and stored at − 80 °C until laboratory measurements were performed. Plasma levels of insulin, glucose, alanine transaminase (ALT), aspartate transaminase (AST), creatine kinase (CK), high-sensitive C-reactive protein (hs-CRP), and plasma lipid patterns (total cholesterol, triglycerides, and HDL-cholesterol) were assessed using standard methods. Insulinemia and glycemia were used to calculate insulin resistance using the homeostatic model assessment index of insulin resistance (HOMA-IR). LDL-cholesterol levels were calculated using the Friedewald formula. Commercial ELISA kits were employed to measure plasma CETP and leptin concentrations (47-CETHU-E01, ALPCO, Salem, NH, USA, and DLP00, R&D Systems Inc., Minneapolis, USA, respectively). Relative fatty acid (FA) contents in erythrocyte membranes were determined using gas-chromatography flame-ionization-detection (GC6850 Agilent Technologies), as previously reported [51]. Red blood cell membrane levels of FAs were expressed as a percent ratio between the AUC of each FA peak and the sum of all FA peaks. Elongase and Desaturase index were calculated as product-to-precursor FA ratio in erythrocyte membrane as follow: Δ-5 desaturase index (Arachidonic/ Dihomo-γ-linolenic ratio); Δ-6 desaturase index (Dihomo-γ-linolenic/Linoleic acid ratio) Δ-9 desaturase index (Palmitic/Palmitoleic acid ratio); Elongase (Stearic/Palmitic ratio). The omega-3 index, defined as the sum of eicosapentaenoic and docosahexaenoic (DHA) acids in erythrocyte membranes, served as a marker of cardiovascular risk.

### Data Presentation and Statistics

To assess the effects of different hormones, volunteers were divided into four groups based on substance abuse, as determined by anonymous questionnaires, in addition to a control group (CTRL). A group of bodybuilders that uses only AAS were grouped in BB_AAS_ (*n* = 13); those who used insulin or in association with AAS alone or AAS plus GH were grouped in BB_INS_ (*n* = 15); those who used GH alone or in association with AAS were grouped in BB_GH_ (*n* = 12); those who were not abusing illicit substances were grouped in BB_NU_ (*n* = 52); and non-bodybuilders were grouped in CTRL (*n* = 45). Data were presented as mean ± SEM. One-way ANOVA with Bonferroni correction post hoc analysis was used to compare results among the four groups of bodybuilders and the control group. Data were log-transformed as necessary to meet normal distribution. P-values < 0.05 were considered statistically significant. Pearson’s correlation test was employed to investigate associations between variables. To address concerns about the limited population using drugs, we also calculated the effect size (θ) to validate significance. Effect size was classified as < 0.2 low probability; 0.2–0.8 medium probability; > 0.8 strong probability. All analyses were conducted using Statistical Package for Social Sciences (SPSS) 21.0 software (SPSS Chicago, IL).

## Results

Results of anonymous questionnaires indicated that 40 out of 92 bodybuilders were using illicit hormones. AAS were taken by 38 over 40 hormone-abusing bodybuilders. Most of them were also using insulin (38%) and/or GH (30%). Details on the type and dosages of insulin used, as well as the consumption of oral supplements, are reported in the supplementary material (Tables 1, 2, 3, 4 and 5). The five groups of participants were well matched for age even though controls were slightly but significantly younger than the BB_NU_ group. Moreover, the four groups of BB were well matched for hours of training (Table 1).

**Table 1:** Characteristics of hormone user bodybuilders, non user bodybuilders and controls

	BB_INS_ (*n* = 15)	BB_GH_ (*n* = 12)	BB_AAS_ (*n* = 13)	BB_NU_ (*n* = 52)	CTRL (*n* = 45)	One-way ANOVA p (η^2^)	Effect Size^*^
Age (year)	28 ± 2	26 ± 1	28 ± 1	32 ± 1^e^	25 ± 1^d^	0.01	0.1 (-0.4–0.6)
Training hours/week	10 ± 1	9 ± 1	7 ± 1	8 ± 1	-	0.48	-
Height (cm)	181.6 ± 1.2	180.1 ± 1.7	180.6 ± 1.5	181.0 ± 0.7	180.7 ± 1.1	0.97	-
Weight (kg)	98.2 ± 3.7^d,e^	93.1 ± 4.9^d,e^	89.6 ± 4.2^e^	84.3 ± 1.7^a,b^	80.0 ± 2.1^a,b,c^	< 0.001	1.0 (0.4–1.5)
BMI (kg/m^2^)	29.7 ± 1.0^d,e^	28.6 ± 1.2^d,e^	27.4 ± 1.2^e^	25.6 ± 0.4^a,b^	24.4 ± 0.5^a,b,c^	< 0.001	1.1 (0.5–1.6)
Fat mass (%)	11.8 ± 1.0^e^	10.9 ± 0.5^e^	10.9 ± 0.4^e^	12.7 ± 0.5^e^	16.0 ± 0.8^a,b,c,d^	< 0.001	-0.4 (-0.9–0.15)
Fat free mass (%)	88.2 ± 1.0^e^	89.1 ± 0.5^e^	89.1 ± 0.4^e^	87.3 ± 0.5^e^	84.0 ± 0.8^a,b,c,d^	< 0.001	0.4 (-0.15–0.9)
Fat free mass index (kg/m^2^)	26.2 ± 0.8^d,e^	25.5 ± 1.0^d,e^	24.4 ± 1.0^d,e^	22.3 ± 0.3^a,b,c,e^	20.4 ± 0.3^a,b,c,d^	< 0.001	1.3 (0.8–1.9)
Vastus lateralis contraction time (ms)	24 ± 1^d,e^	23 ± 0^d,e^	23 ± 1^d,e^	28 ± 1^a,b,c^	29 ± 1^a,b,c^	< 0.001	-0.7 (-1.3 - -0.05)
Biceps femoris contraction time (ms)	25 ± 1^b,e^	21 ± 1^a,c,d,e^	26 ± 2^b,e^	27 ± 1^b,e^	32 ± 1^a,b,c,d^	< 0.001	-0.2 (-0.8–0.4)

Values are expressed as mean ± SEM. BB_INS_, bodybuilders abusing insulin alone (*n* = 1) or in association with anabolic androgenic steroids (*n* = 3) or with anabolic androgenic steroids and growth hormone (*n* = 11); BB_GH_, bodybuilders abusing growth hormone alone (*n* = 1) or in association with anabolic androgenic steroids (*n* = 11); BB_AAS_, bodybuilders abusing only anabolic androgenic steroids; BB_NU_, bodybuilders not using hormones; CTRL, non-bodybuilders. Statistical analysis was performed using One-way ANOVA with Bonferroni post hoc analysis

a, significant difference from BB_INS_ (*P* < 0.05); b, significant difference from BB_GH_ (*P* < 0.05); c, significant difference from BB_AAS_ (*P* < 0.05); d, significant difference from BB_NU_ (*P* < 0.05); e, significant difference from CTRL (*P* < 0.05).

*Effect size is calculated to quantify differences between BB_INS_ group versus all the other groups. If post-hoc analysis showed a difference between BB_INS_ and more than one group, both pooled average and standard deviation of these groups were considered to assess effect size. < 0.2 low probability; 0.2–0.8 medium probability; > 0.8 strong probability. Effect size is expressed as point estimate and confidence intervals (95%)

Body composition, anthropometric measurements, and TMG parameters are reported in Table 1. All bodybuilders’ groups displayed lower percent of FM and higher percent of FFM than CTRL while the FFMi was significantly higher in bodybuilders using hormones when compared to the non-hormone users or CTRL, while FFMi was found higher in BB_NU_ as compared to CTRL.

Tc of VL was significantly shorter in hormone user bodybuilders than in non-hormone user bodybuilders and/or CTRL (Table 1). The shortest Tc of BF was observed in BB_GH_ and the longest in CTRL.

Table 2 reports results from metabolic markers evaluation. Hormonal abuse did not significantly affect fasting plasma insulin and glucose concentrations. Bodybuilding with or without hormone abuse did not significantly affect plasma triglycerides and LDL-cholesterol, while non-hormonal user bodybuilders and CTRL showed significantly higher HDL-cholesterol level. Such an increase seems to be abolished by hormone intake, especially when combined (BB_INS_ and BB_GH_). Plasma concentrations of CETP were similar in controls and non-hormone user bodybuilders or in selective AAS abuse, while they were significantly lower in BB_GH_ group. The BB_INS_ group, which includes 73% of bodybuilders using also GH, did not show significant differences in CETP levels when compared to BB_GH_ group (not using insulin). Pooled GH users (*n* = 23) exhibited significantly lower CETP levels as compared to all other non-GH user (*n* = 69) bodybuilders (2.68 ± 0.03 versus 3.12 ± 0.01 mg/mL; *p* = 0.02).

**Table 2:** Effects of bodybuilding and hormone abuses on metabolic markers

	BB_INS_ (*n* = 15)	BB_GH_ (*n* = 12)	BB_AAS_ (*n* = 13)	BB_NU_ (*n* = 52)	CTRL (*n* = 45)	One-way ANOVA p (η^2^)	Effect Size*
INSULIN SENSITIVITY							
Fasting glucose (mg/dL)	90 ± 2	87 ± 4	89 ± 2	93 ± 1	91 ± 1	0.15	-
Fasting insulin (µU/mol)	7.2 ± 1.1	11.0 ± 4.9	7.1 ± 1.0	6.3 ± 0.4	8.3 ± 0.9	0.22	-
HOMA index	1.61 ± 0.25	2.38 ± 1.03	1.59 ± 0.24	1.46 ± 0.11	1.90 ± 0.22	0.30	-
**PLASMA LIPID PATTERN**							
Total cholesterol (mg/dL)	168.6 ± 10.2^b^	139.7 ± 14.4^a,c,d^	179.3 ± 13.1^b^	173.7 ± 4.8^b,e^	157.2 ± 4.6^d^	0.02	0.1 (-0.4–0.6)
Triglycerides (mg/dL)	99.8 ± 18.8	79.2 ± 13.0	88.7 ± 16.9	101.5 ± 14.5	75.6 ± 6.3	0.52	-
HDL-cholesterol (mg/dL)	31.3 ± 3.7^c,d,e^	27.1 ± 3.3^c,d,e^	37.8 ± 2.5^a,b,d^	48.0 ± 1.5^a,b,c,e^	42.0 ± 1.2^a,b,d^	< 0.001	-0.9 (-1.5 - -0.4)
LDL cholesterol (mg/dL)	117.3 ± 9.5	96.8 ± 11.5	123.8 ± 13.8	105.4 ± 3.9	100.0 ± 3.6	0.08	0.4 (-0.1–0.9)
HDL-to-non-HDL cholesterol ratio	0.25 ± 0.04^d,e^	0.28 ± 0.04^d,e^	0.30 ± 0.04^d^	0.42 ± 0.02^a,b,c^	0.39 ± 0.03^a,b^	0.001	-0.8 (-1.3 - -0.2)
**OTHER PLASMA MARKERS**							
CETP (µg/mL)	2.76 ± 0.10^c^	2.57 ± 0.25^c,d,e^	3.45 ± 0.22^a,b^	3.08 ± 0.11^b^	3.16 ± 0.10^b^	0.02	-0.4 (-1.0–0.09)
Leptin (ng/mL)	1.22 ± 0.46^e^	0.61 ± 0.18^e^	0.63 ± 0.18^e^	0.86 ± 0.12^e^	2.69 ± 0.51^a,b,c,d^	< 0.001	-0.1 (-0.7–0.4)
Leptin/kg of fat (ng/mL*kg)	0.09 ± 0.03^e^	0.06 ± 0.02^e^	0.05 ± 0.01^e^	0.08 ± 0.01^e^	0.18 ± 0.02^a,b,c,d^	< 0.001	-0.2 (-0.8–0.3)
Creatinine (mg/dL)	1.22 ± 0.04	1.14 ± 0.05	1.17 ± 0.04	1.17 ± 0.02	1.18 ± 0.02	0.70	-
ALT (I.U./L)	66.7 ± 10.0^b,c,d,e^	46.8 ± 7.1^a,c,d,e^	31.2 ± 3.3^a,b,e^	26.5 ± 1.7^a,b,e^	22.0 ± 1.3^a,b,c,d^	< 0.001	2.1 (1.5–2.7)
AST (I.U./L)	53.9 ± 11.0^c,d,e^	54.6 ± 10.0^c,d,e^	29.9 ± 3.9^a,b^	28.2 ± 1.4^a,b,e^	23.2 ± 1.2^a,b,d^	< 0.001	1.2 (0.6–1.8)
ALT/AST	1.41 ± 0.14^b,c,d,e^	0.96 ± 0.09^a^	1.10 ± 0.11^a^	0.97 ± 0.04^a^	0.97 ± 0.04^a^	0.001	1.2 (0.6–1.8)
CK (U./L.)	951 ± 343^c,d,e^	966 ± 257^c,d,e^	356 ± 91^a,b,e^	302 ± 40^a,b,e^	180 ± 31^a,b,c,d^	< 0.001	1.4 (0.6–2.1)
hs-CRP (mg/dL)	0.16 ± 0.06^d,e^	0.16 ± 0.07	0.14 ± 0.05^d^	0.07 ± 0.02^a,c^	0.08 ± 0.01^a^	0.01	0.5 (-0.03–1.0)

Values are expressed as mean ± SEM. BB_INS_, bodybuilders abusing insulin alone (*n* = 1) or in association with anabolic androgenic steroids (*n* = 3) or with anabolic androgenic steroids and growth hormone (*n* = 11); BB_GH_, bodybuilders abusing growth hormone alone (*n* = 1) or in association with anabolic androgenic steroids (*n* = 11); BB_AAS_, bodybuilders abusing only anabolic androgenic steroids; BB_NU_, bodybuilders not using hormones; CTRL, non-bodybuilders

Statistical analysis was performed using One-way ANOVA with Bonferroni post hoc analysis. a, significant difference from BB_INS_ (*P* < 0.05). b, significant difference from BB_GH_ (*P* < 0.05). c, significant difference from BB_AAS_ (*P* < 0.05). d, significant difference from BB_NU_ (*P* < 0.05). e, significant difference from CTRL (*P* < 0.05)

*Effect size is calculated to quantify differences between BB_INS_ group versus all the other groups. If post-hoc analysis showed a difference between BB_INS_ and more than one group, both pooled average and standard deviation of these groups were considered to assess effect size. < 0.2 low probability; 0.2–0.8 medium probability; > 0.8 strong probability. Effect size is expressed as point estimate and confidence intervals (95%)

HOMA-IR, homeostatic model assessment index of insulin resistance. CETP, Cholesteryl ester transfer protein. ALT, alanine transaminase. AST, aspartate transaminase. CK, creatine kinase hs-CRP, high-sensitive C-reactive protein

Plasma leptin concentrations were significantly decreased in all bodybuilders as compared to controls. In all volunteers (*n* = 147), the values of leptin concentration directly correlated with FM (kg) (*r* = 0.70, *p* < 0.001). The leptin-to-FM ratio was significantly decreased in all groups of bodybuilders as compared to the CTRL group. Moreover, we found two direct correlations: one between leptin-to-FM ratio and insulinaemia (*R* = 0.32; *p* < 0.001) and another between leptin-to-FM ratio and HOMA-IR index (*r* = 0.34; *p* < 0.001). BB_INS_ and BB_GH_ showed no differences in CK levels while they presented three times higher CK concentration when compared to BB_AAS_ and 3/4 times greater than BB_NU_ or controls. The BB_NU_ group showed higher CK levels only when compared to controls.

ALT and AST concentrations were significantly higher in BB_INS_ and BB_GH_ bodybuilders than those observed in all other groups. ALT concentrations were further increased in insulin-user bodybuilders, while AST is similar between BB_INS_ and BB_GH_ groups. The ALT-to-AST ratio was significantly greater in insulin-user bodybuilders than in all other groups. CK concentrations directly correlated with ALT (*r* = 0.31; *p* < 0.001) but not with AST or the ALT-to-AST ratio. hs-CRP was greater in hormone user bodybuilders that in non-hormone users plus controls (*p* < 0.005).

The complete pattern of red blood cell membrane FA composition as well as the estimated elongase and desaturase enzyme activities are shown in Table 3. Insulin-users exhibited significantly higher Δ-9 desaturase-16 activity compared to all the other groups while, elongase activity was lower in insulin and/or growth hormone doping.

**Table 3:** Erythrocyte membrane fatty acid composition and activity of enzymes involved in fatty acid metabolism in recruited male subjects, divided depending on hormone abuse

	BB_INS_ (*n* = 15)	BB_GH_ (*n* = 12)	BB_AAS_ (*n* = 13)	BB_NU_ (*n* = 52)	CTRL (*n* = 45)	One-way ANOVA	Effect Size*
SATURATED FAs							
Myristic 14:00	0.31 ± 0.03	0.30 ± 0.03	0.28 ± 0.02	0.33 ± 0.01	0.35 ± 0.02	0.06	-0.2 (-0.8–0.3)
Palmitic 16:00	23.18 ± 0.55^c,d^	23.18 ± 0.62^c,d^	21.49 ± 0.40^a,b^	20.79 ± 0.15^a,b,e^	22.15 ± 0.42^d^	< 0.001	0.7 (0.18–1.3)
Stearic 18:00	16.33 ± 0.50^d,e^	16.15 ± 0.40^c,d,e^	17.31 ± 0.35^b,e^	18.01 ± 0.12^a,b,e^	19.31 ± 0.23^a,b,c,d^	< 0.001	-1.2 (-1.7 - -0.6)
SUM	39.79 ± 0.41 ^e^	39.60 ± 0.38^e^	39.05 ± 0.50^e^	39.13 ± 0.15^e^	41.82 ± 0.61^a,b,c,d^	0.001	-0.1 (-0.7–0.4)
**MONOUNSATURATED FAs**							
Palmitoleic 16:1n-7	0.33 ± 0.06^c,d,e^	0.25 ± 0.05	0.19 ± 0.03^a^	0.21 ± 0.01^a^	0.23 ± 0.01^a^	0.01	1.0 (0.45–1.5)
Oleic 18:1n-9	13.90 ± 0.43	13.56 ± 0.42	12.95 ± 0.23	13.23 ± 0.16	13.68 ± 0.26	0.26	-
Eicosaenoic 20:1n-9	0.28 ± 0.02^b,c,d,e^	0.23 ± 0.02^a^	0.22 ± 0.02^a^	0.24 ± 0.01^a^	0.23 ± 0.01^a^	0.02	0.5 (0.001–1.0)
SUM**	15.77 ± 0.46	15.42 ± 0.49	14.62 ± 0.28	14.81 ± 0.16	15.37 ± 0.29	0.12	-
**n-3 POLYUNSATURATED FAs**							
α-Linolenic 18:3n-3	0.13 ± 0.01^e^	0.11 ± 0.03^e^	0.11 ± 0.02^e^	0.12 ± 0.01^e^	0.30 ± 0.03^a,b,c,d^	< 0.001	-0.5 (-1.0–0.08)
Eicosapentaenoic 20:5n-3	1.80 ± 0.38^b,c,d,e^	1.28 ± 0.29^a,c,d,e^	0.62 ± 0.16^a,b^	0.72 ± 0.04^a,b,e^	0.45 ± 0.03^a,b,d^	< 0.001	1.692 (1.1–2.2)
Docosapentaenoic 22:5n-3	2.69 ± 0.18^c,e^	2.62 ± 0.17^e^	2.31 ± 0.13^a,d^	2.72 ± 0.04^c,e^	2.30 ± 0.07^a,b,d^	< 0.001	0.3 (-0.2–0.9)
Docosahexaenoic 22:6n-3	5.30 ± 0.38^e^	5.03 ± 0.41	4.70 ± 0.43^d^	5.52 ± 0.16^c,e^	4.28 ± 0.20^a,d^	< 0.001	0.3 (-0.3–0.8)
SUM	9.85 ± 0.80^c,e^	9.03 ± 0.79^e^	7.71 ± 0.62^a,d^	9.07 ± 0.21^c,e^	7.18 ± 0.27^a,b,d^	< 0.001	0.7 (0.2–1.3)
ω-3 index	7.10 ± 0.67^c,e^	6.31 ± 0.66^e^	5.32 ± 0.55^a^	6.24 ± 0.19^e^	4.71 ± 0.22^a,b,d^	< 0.001	0.9 (0.27–1.36)
**n-6 POLYUNSATURATED FAs**							
Linoleic 18:2n-6	12.71 ± 0.59^b,d^	14.09 ± 0.83^a,d,e^	13.01 ± 0.66^d,e^	11.64 ± 0.20^a,b,c^	11.77 ± 0.19^b,c^	< 0.001	0.3 (-0.2–0.9)
Eicosadienoic 20:2n-6	0.65 ± 0.23	0.43 ± 0.12	0.68 ± 0.23	0.50 ± 0.07	0.40 ± 0.03	0.34	-
Dihomo-γ-linolenic 20:3n-6	1.53 ± 0.15^d^	1.67 ± 0.17^d^	1.69 ± 0.14^d^	2.00 ± 0.08^a,b,c,e^	1.73 ± 0.06^d^	0.01	-0.5 (-1.1 - -0.03)
Arachidonic 20:4n-6	15.44 ± 0.57^c,d^	15.74 ± 0.46^c,d^	17.68 ± 0.32^a,b^	17.70 ± 0.26^a,b^	16.79 ± 0.45	< 0.01	-0.7 (-1.3 - -0.2)
Adrenic 22:4n-6	2.88 ± 0.26^c,d,e^	2.98 ± 0.32^c,d,e^	3.73 ± 0.23^a,b^	3.79 ± 0.09^a,b^	3.98 ± 0.15^a,b^	< 0.001	-1.0 (-1.5 - -0.4)
Docosapentaenoic 22:5n-6	0.39 ± 0.04^c,d,e^	0.44 ± 0.05^c,d,e^	0.61 ± 0.06^a,b,e^	0.67 ± 0.03^a,b^	0.74 ± 0.03^a,b,c^	< 0.001	-1.2 (-1.7 - -0.6)
SUM	33.64 ± 0.86^c,d^	35.38 ± 0.99	37.42 ± 0.67^a^	36.31 ± 0.31^a^	35.39 ± 0.67	0.03	-0.9 (-1.4 - -0.4)
**ENZYME ACTIVITIES**							
Δ9 desaturase (16:1/16:0)	0.014 ± 0.002^b,c,d,e^	0.011 ± 0.002^a^	0.009 ± 0.001^a^	0.010 ± 0.001^a^	0.010 ± 0.000^a^	0.03	0.9 (0.3–1.4)
Δ5 desaturase (20:4n6/20:3n6)	11.66 ± 1.4	10.44 ± 1.1	11.46 ± 1.1	9.57 ± 0.4	10.2 ± 0.5	0.26	-
Elongase (18:0/16:0)	0.72 ± 0.04c,d,e	0.71 ± 0.04c,d,e	0.81 ± 0.02a,b,d,e	0.87 ± 0.01a,b,c	0.88 ± 0.01a,b,c	< 0.001	-

Values are expressed as mean (%) ± SEM. BB_INS_, bodybuilders abusing insulin alone (*n* = 1) or in association with anabolic androgenic steroids (*n* = 3) or with anabolic androgenic steroids and growth hormone (*n* = 11); BB_GH_, bodybuilders abusing growth hormone alone (*n* = 1) or in association with anabolic androgenic steroids (*n* = 11); BB_AAS_, bodybuilders abusing only anabolic androgenic steroids; BB_NU_, bodybuilders not using hormones; CTRL, non-bodybuilders

Statistical analysis was performed by using One-way ANOVA with Bonferroni post hoc analysis. a, significant difference from BB_INS_ (*P* < 0.05). b, significant difference from BB_GH_ (*P* < 0.05). c, significant difference from BB_AAS_ (*P* < 0.05). d, significant difference from BB_NU_ (*P* < 0.05). e, significant difference from CTRL (*P* < 0.05)

*Effect size is calculated to quantify differences between BB_INS_ group versus all the other groups. If post-hoc analysis showed a difference between BB_INS_ and more than one group, both pooled average and standard deviation of these groups were considered to assess effect size. < 0.2 low probability; 0.2–0.8 medium probability; > 0.8 strong probability. Effect size is expressed as point estimate and confidence intervals (95%)

**The monounsaturated sum had been calculated including elaidic acid content (not reported in table)

Δ9 desaturase activity was estimated as as palmitoleic-to-palmitic acid ratio. Δ5 desaturase activity was estimated as arachidonic-to-dihomo-γ-linolenic ratio acid ratio. Elongase activity was estimated as stearic-to-palmitic ratio

**Fig. 1:**
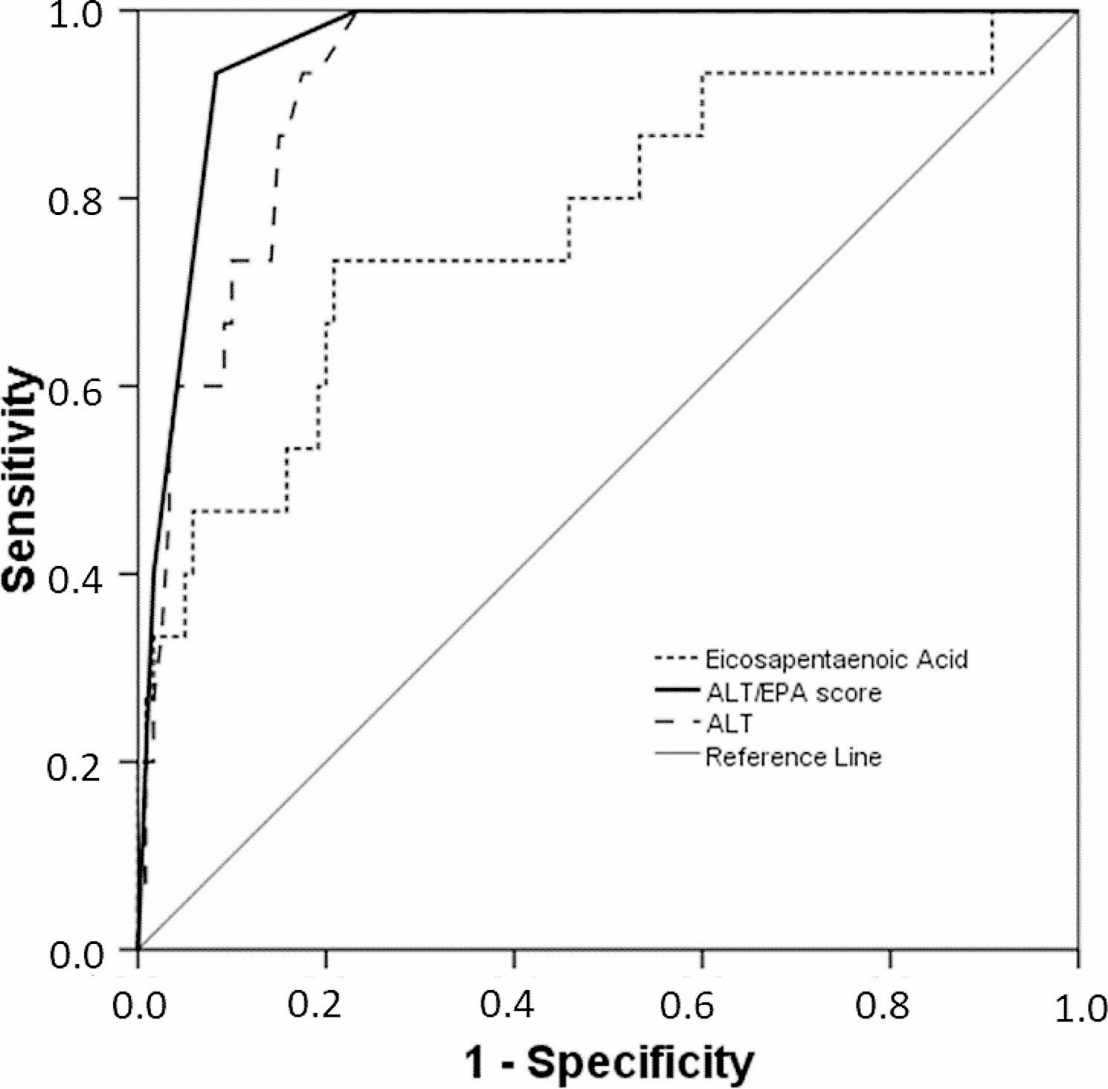
ROC curve of the score to identify insulin doping consumption

Furthermore, trying to obtain the best predictors of insulin abuse by using common blood analysis, ALT and eicosapentaenoic acid reduction have been combined in a score. The best combination is obtained adding ALT level (0 if lower than 32.5 mU/mL, 1 if between 32.5 and 60 and 2 if above 60), eicosapentaenoic (0 if lower than 0.8106% and 1 if above) into a digit ranging from 0 to 4. Such a score, in ROC curve having insulin use as discriminant variable, gave an AUC of 0.959 (SE 0.017; 95% C.I. 0.927–0.992; *p* < 0.001). The curve is reported in Fig. 1. Dichotomizing the subjects in those with none or 1 positive result Vs those with two or three, only one false negative and ten false positive could be identified (HR 157; 95% C.I. 19-1319; *p* < 0.001). The results are reported in Table 4.

**Table:** 

Variables	Area	St. Error	Significance	95% Confidence Interval	
Eicosapentaenoic acid	0.774	0.071	0.001	0.634	0.914
ALT + EPA Score	0.959	0.017	0.001	0.927	0.992
ALT	0.930	0.024	0.001	0.884	0.976

**Table 4:** 2 × 2 crosstab of the score dichotomized and insulin consumption in bodybuilders

		bodybuilders		Total
		Non-insulin consumption	Insulin consumption	
Score	0 or 1	112	1	113
	2 or 3	10	14	24
Total		122	15	137

## Discussion

Hormone abuse is a widespread illegal practice in both professional and recreational sports. Among the hormones commonly abused in sports and physical activity, androgenic anabolic steroids (AASs) are the most prevalent, reaching 95% in our group. However, over the past two decades, insulin and human-recombinant GH have emerged as new substances widely introduced into sports doping. As confirmed also by our study, AASs remain the most abused drug among bodybuilders. Nevertheless, the misuse of GH and insulin is similarly significant, involving 25% and 16% of the subjects recruited in our study, respectively. Among these hormones, only the injection of exogenous human recombinant insulin currently escapes detection methods [39].

As expected, engaging in resistance training resulted in an increase in the FFM and a coincident decrease in FM. Fat mass secretes plasma leptin [52], this biomarker reflects the amount of adipose stores and is further regulatedby meals [53]: fasting causes a rapid initial decrease in circulating leptin levels that becomes more marked with progressive body fat loss [54]. Consistently, our recreational bodybuilders exhibited low leptin levels and fat mass compared to the control group. The lack of dietary habits record prevents from a more precise picture of such a relationship. The other hormones used by our Body builders do not have any significant impact on fat mass or leptin concentration while AAS, either alone or in combination with GH and/or insulin and as previously observed [55, 56], is associated with higher FFM. Consistent with other studies, hormone abuse does not affect body composition [46]: beyond higher FFM index among subjects who abused prohibited substances, the combinations of AAS with GH and/or insulin did not exhibit any additional or synergistic effects on this marker. Therefore, in line with previous research findings [53, 57], our data indicates that only AAS, and not insulin or GH, clearly demonstrates anabolic properties in healthy, active individuals.

### Body Composition and Muscle Function

TMG assessing muscle contractile time during maximum electrically induced isometric twitch contractions [8, 49, 50]. The contraction time measured through TMG depends on the composition of muscle fiber types [8, 49, 50]. Postural Vastus Lateralis and non-postural Biceps Femoris show a similar but not identical pattern of response during hormonal abuse, with the former being significantly shorter for all abusers compared to controls and non-users and the latter being shorter for all bodybuilders but having a significantly shorter time for those assuming growth hormone and insulin. The explanation might be in the increased number of type 2 fibers deriving from bodybuilding itself [7] further enhanced by GH and Insulin use [18, 58] and the increase of both type 1 and 2 fibers from users of steroids [59]. The putative decrease in the prevalence of type-1 fibers could also induce insulin resistance [58], possibly through a reduction in the GLUT-4 pool [60]. The contraction time and muscle fiber composition in all BB using drugs suggest that there are no evident additive or synergistic effects from hormones combination and, if that, it might depend on muscle type.

### Glucose Metabolism

In contrast with previous observations demonstrating that in healthy subjects insulin [61], GH [23] and AAS [62] administration impairs insulin sensitivity, this marker was comparable among all groups. Only in subjects assuming GH, insulin level and HOMA index showed a trend towards higher values. This surprising normal insulin sensitivity in bodybuilders abusing illicit hormones might have several explanations: constant and regular physical exercise prevents insulin resistance, the HOMA index could not be a suitable marker of insulin sensitivity in subjects with very high ratio between muscle and fat mass and, last, the doping-induced lower amount of insulin-sensitive type-1 fibers in bodybuilders.

In agreement with previous observations in elite weightlifters [63], leptin levels, normalized for fat mass, were lower in bodybuilders compared to controls. Insulin is a factor known to enhance leptin availability [64] and we found a direct correlation between leptin-to-FM ratio and insulinemia and another between leptin-to-FM ratio and HOMA-IR index. Nonetheless, in our study, leptin levels were slightly and not significantly increased in insulin-user bodybuilders.

### Liver Function

ALT and AST circulating levels are sensitive markers of hepatocyte damage (cytolysis) although not specific: skeletal and myocardial muscle damage contribute to their levels, especially when associated to high CK levels. In our study, resistant training is associated with higher ALT, AST, and CK levels, particularly in BB assuming insulin and GH. CK level directly and significantly correlates to both transaminase contents, suggesting that, in bodybuilders, muscle damage, more than liver dysfunction accounts for such an increase. Muscle damage in our bodybuilders can be related to higher resistant training [65] as well as to repeated hormone subcutaneous injections [66]. However, the ALT-to-AST ratio, which is a specific marker of liver damage [67], was selectively increased in our insulin abusing bodybuilders and did not correlate with CK. This suggests that the observed increase in ALT-to-AST ratio could be due to a direct insulin action on liver, possibly associated with increased lipid synthesis and fatty acid infiltration [15]. Thus, an ALT-to-AST ratio could represent a selective marker of insulin abuse in bodybuilders, although, further investigations are required to validate our hypothesis.

### Inflammatory Markers

Exercise training is classically associated with decreased systemic inflammation and CRP levels, being strength training less effective on CRP levels than aerobic training [68]. In our study, subjects abusing insulin and AAS, displayed an increase in hs-CRP content. The same pattern, although marginally significant (*p* = 0.057) was found in subjects abusing GH. Mechanisms throughout which hormones trigger hs-CRP.

### Lipid Metabolism

Membrane FA composition reflects long term dietary habits [51, 69–73]. Our data shows that enrolled bodybuilders consumed lower amounts of saturated FAs and higher quantity of n-3 FAs, with the exception of bodybuilders abusing AAS. As consequence of n-3 supplementation, lower membrane n-6 FA content was confirmed in bodybuilders. The composition of fatty acids in red blood cells may hold significance, particularly in light of the observed association between an increase in saturated fatty acids and insulin resistance [74–76]. In humans, concentrations of monounsaturated fatty acids (MUFA) in muscle phospholipids have shown a positive correlation with fasting plasma insulin levels but a negative correlation with the presence of polyunsaturated fatty acids (PUFA) in muscle content [75]. The changes in fatty acid composition within the lipid membranes of skeletal muscle in insulin-resistant, obese, or diabetic individuals, both in rodents and humans, could be attributed to an altered pattern of fatty acid synthesis. This switch may derive from changes in enzyme activities responsible for elongation and desaturation of fatty acids [77], potentially occurring in the liver with subsequent transport to skeletal muscle. Alternatively, it might be due to a local shift in the fatty acid synthesis pattern, although it is generally believed that de novo lipogenesis rates in skeletal muscle are low in both humans and experimental animals [78]. To create long-chain monounsaturated fatty acids (MUFA) and polyunsaturated fatty acids (PUFA), fatty acids undergo desaturation and elongation processes with the assistance of Δ5-, Δ6-, and Δ9-desaturases, which introduce double bonds at the fifth, sixth, and ninth carbon from the carboxyl terminal, respectively [79, 80]. Elongation is accomplished through the action of the ubiquitous elongase, which adds two carbon units at the carboxyl terminal of fatty acids [77]. To define whole body status, the activity of enzymes involved in FA metabolism, estimated as product-to-precursor ratio, was evaluated, independently from volunteers’ dietary habits. Metabolic regulations of Δ-9 desaturase and elongase expressions are strictly, although negatively, correlated. These enzymes are induced by insulin and glucose, through regulation of specific transcription factors (i.e., SREBP-1, ChREBP and MLX) involved in fatty acid metabolism [81]. In fact, it was demonstrated that Insulin exerts its effect on desaturase expression through SREBP-1c both at transcriptional, post-transcriptional and translational levels. SREBP-1c is an important candidate in mediating the effects of insulin on desaturase gene expression, linking nutritional pathways of carbohydrates, insulin metabolic reply and lipid metabolism [82]. In vitro studies demonstrate that Δ-9 desaturase deficiency selectively stimulates elongase expression, whereas an increase in monounsaturated FAs inhibits elongase expression through a negative-feedback mechanism [83]. The balance between Δ-9 desaturase and elongase-6 activities, observed also in our bodybuilders assuming insulin and GH, could aim at preservation of cell membrane activities and functions through the maintenance of proper amount of specific monounsaturated FAs, as previously suggested [83]. Even though the cause-effect relationship are still unclear, Δ-9 desaturases and elongase activities have been previously related to several metabolic risk factors [84]. In our study, bodybuilders abusing insulin and GH, have low elongase-6 and high Δ-9 desaturase activities, low HDL levels as well as a higher hs-CRP concentration. On the contrary, in bodybuilders abusing AAS, with normal HDL-cholesterol, the elongase activity was impaired at a lesser extent than in subjects abusing insulin and/or GH. Delta-9 desaturase activity was selectively higher and elongase activity was reduced in insulin-user bodybuilders, triggering the hypothesis that eicosapentaenoic acid increased in insulin consumers is the final product of such an imbalance. Even though further detailed studies are required, it looks like insulin is a factor inducing the synthesis of shorter and unsaturated lipids. As a matter of fact, eicosapentaenoic acid, more than Δ-9 desaturase and elongase, represents another selective marker of insulin abuse in the present study.

### Limitations of the Study

Studies aiming to identify the anabolic effects and side effects of doping have typically faced ethical limitations, particularly concerning the quality and quantity of hormone administrations. Abuse of illicit substances used in doses that are beyond what can be administered in interventional research studies involving healthy subjects is a major issue in sports. The strength of our study lies in its ability to monitor and investigate hormonal doping within a community of bodybuilders, free from external interference related to the quantity and quality of hormone abuse. Nonetheless, it is essential to acknowledge some limitations of this observational study: (1) the small sample sizes may limit the ability to detect small effect sizes and can lead to overestimation or underestimation of effect sizes. These limitations could affect the reliability of some findings, when generalized to larger populations or other settings; (2) details about AAS and GH abuse are missed (specific compounds, dosage, use duration and administration via). It is also important to point out that AAS administration (via injection or oral route) may have different effects on metabolic parameters [85, 86]; (3) results should be reevaluated and confirmed by a cohort study. In turn, cohort studies are challenging due to a difficult tight control of subject compliance and truthfulness in anonymously completed questionnaires which remain a big obstacle with every type of study design; (4) The study lacks data about dietary habits. Dietary choices and nutritional intake can significantly influence metabolic parameters, body composition, and overall health. Macronutrient composition, calorie intake, and meal timing, play a crucial role in glucose metabolism and lipid metabolism. These factors can affect insulin sensitivity, lipid profiles, and inflammatory markers. To enhance the robustness of future investigations, comprehensive data on dietary habits should be collected. This would involve assessing participants’ daily caloric intake, macronutrient distribution, meal frequency, and specific food choices. Understanding the interplay between hormonal abuse and diet might provide a more multifaceted interpretation of study outcomes.

## Conclusions

Insulin and GH abuse is associated with multiple modifications of specific metabolic markers. In these subjects, we observed a decrease in HDL levels that was paralleled by a decrease in CETP availability. In these same subjects, activities of Δ-9 desaturase and elongase, enzymes considered predictor of metabolic risks, were varied. Moreover, insulin abuse is peculiarly characterized by high ALT-to-AST ratio and Δ-9 desaturase activity. These findings might help in the development of selective and sensitive markers for longitudinal doping detection, which could be incorporated into the Athlete Biological Passport.

Even though further studies are required, we suggest that longitudinal monitoring of selected metabolic markers such as muscle contraction time, HDL and CETP levels, ALT-to-AST ratio as well as activities of selected enzymes involved in membrane fatty acid metabolism (i.e. Δ-9 desaturase and elongase), could contribute to detection of insulin and GH abuse in sport.

### Electronic Supplementary Material

Below is the link to the electronic supplementary material.

Supplementary Material 1

## Data Availability

Data will be made available for a reasonable request.

## Abbreviations

AAS: Anabolic Androgenic Steroids
GH: Growth Hormone
IGF: 1-Insulin-Like Growth Factor-1
RT: Resistance Training
WADA: World Anti-Doping Agency
FFM: Fat-Free Mass
FM: Fat Mass
BIA: Multifrequency Bioimpedance
FFMi: FFM Index
TMG: Tensiomyography
VL: Vastus Lateralis
BF: Biceps Femoris
ALT: Alanine Transaminase
AST: Aspartate Transaminase
CK: Creatine Kinase
hs: CRP-High-Sensitive C-Reactive Protein
HOMA: IR-Homeostatic Model Assessment Index Of Insulin Resistance
FA: Fatty Acid
DHA: DocosaHexaenoic Acid
