# Supplementary material for: Metabolic Consequences of Anabolic Steroids, Insulin, and Growth Hormone Abuse in Recreational Bodybuilders: Implications for the World Anti-Doping Agency Passport
Source: Sports Med Open. 2024 Mar 27;10:28. doi: 10.1186/s40798-024-00697-6 (PMC10973313; doi:10.1186/s40798-024-00697-6)
Supplement: Supplementary file 1 — Supplementary Material 1 [file 40798_2024_697_MOESM1_ESM.docx]

**SUPPLEMENTARY MATERIAL**

**Sports Medicine – Open Journal**

**METABOLIC CONSEQUENCES OF ANABOLIC STEROIDS, INSULIN, AND GROWTH HORMONE ABUSE IN RECREATIONAL BODYBUILDERS: IMPLICATIONS FOR THE WORLD ANTI-DOPING AGENCY PASSPORT**

Filippo Giorgio Di Girolamo^1,4^, Chiara Biasinutto^4^, Alessandro Mangogna^6^, Nicola Fiotti^1^, Pierandrea Vinci^5^, Rado Pisot^3^, Filippo Mearelli^5^, Bostjan Simunic^2,3^, Chiara Roni^4^, Gianni Biolo^1,5^

1. Department of Medical, Surgical and Health Sciences, University of Trieste – Trieste, Italy.
2. University of Primorska, Koper, Slovenia.
3. Science and Research Centre Koper, Institute for Kinesiology Research, Koper, Slovenia.
4. SC Assistenza Farmaceutica e Territoriale, Azienda Sanitaria Universitaria Giuliano Isontina, – Trieste, Italy.
5. UCO Clinica Medica, Azienda Sanitaria Universitaria Giuliano Isontina, – Trieste, Italy.
6. Institute for Maternal and Child Health, Istituto di Ricovero e Cura a Carattere Scientifico (IRCCS) Burlo Garofolo, Trieste, Italy

**Corresponding author:**

**Filippo Giorgio Di Girolamo PharmaD, PhD**

SC Assistenza Farmaceutica e Territoriale, Ospedale di Cattinara,

Strada di Fiume, 447

34149 – Trieste

Italy

Email: fgdigirolamo@units.it

**Table S1. Oral supplements intake**

|  | **BB_INS_**  **(n=15)** | **BB_GH_**  **(n=12)** | **BB_AAS_**  **(n=13)** | **BB_NU_**  **(n=52)** | **CTRL**  **(n=45)** | **TOT**  **(137)** |
| --- | --- | --- | --- | --- | --- | --- |
| Protein | 11 | 6 | 8 | 19 | 0 | 44 |
| Creatin | 12 | 9 | 12 | 27 | 0 | 60 |
| Amino Acids | 8 | 8 | 9 | 21 | 0 | 46 |
| Glutamin | 8 | 3 | 2 | 10 | 0 | 23 |
| Ephedrin | 5 | 0 | 2 | 7 | 0 | 14 |
| Caffeine | 3 | 0 | 1 | 3 | 0 | 7 |
| Arginine | 2 | 0 | 1 | 1 | 0 | 4 |
| Nitric Oxide | 1 | 0 | 2 | 0 | 0 | 3 |
| Vitamins | 4 | 1 | 1 | 6 | 0 | 12 |
| Taurin | 1 | 1 | 0 | 2 | 0 | 4 |
| Mineral salts | 3 | 1 | 0 | 0 | 0 | 4 |
| BCAAs | 2 | 4 | 3 | 9 | 0 | 18 |
| L-carnitines | 1 | 0 | 1 | 1 | 0 | 3 |
| potassium | 0 | 0 | 0 | 1 | 0 | 1 |
| Iron | 1 | 0 | 0 | 1 | 0 | 2 |
| Magnesium | 1 | 0 | 0 | 1 | 0 | 2 |
| HMB | 1 | 0 | 0 | 1 | 0 | 2 |
| Beta-Alanine | 0 | 1 | 0 | 0 | 0 | 1 |
| Ornitin | 0 | 0 | 0 | 1 | 0 | 1 |
| Zink | 0 | 0 | 0 | 1 | 0 | 1 |
| Maltodextrin | 0 | 0 | 0 | 1 | 0 | 1 |
| Omega-3 | 2 | 5 | 2 | 0 | 0 | 9 |

Distribution of oral supplement intake in the population expressed as absolute values. BB_INS_, bodybuilders abusing insulin alone (n=1) or in association with anabolic androgenic steroids (n=3) or with anabolic androgenic steroids and growth hormone (n=11); BB_GH_, bodybuilders abusing growth hormone alone (n=1) or in association with anabolic androgenic steroids (n=11); BB_AAS_, bodybuilders abusing only anabolic androgenic steroids; BB_NU_, bodybuilders not using hormones; CTRL, non-bodybuilders. BCAA, Branched chain amino acids; HMB, β-Hydroxy β-methylbutyric acid.

**Table S2. Other drugs and phytotherapeutics intake**

|  | **BB_INS_**  **(n=15)** | **BB_GH_**  **(n=12)** | **BB_AAS_**  **(n=13)** | **BB_NU_**  **(n=52)** | **CTRL**  **(n=45)** | **TOT**  **(137)** |
| --- | --- | --- | --- | --- | --- | --- |
| IGF-1 | 2 | 1 | 1 | 0 | 0 | 4 |
| Testosteron | 1 | 0 | 0 | 0 | 0 | 1 |
| Liothyronine | 0 | 1 | 0 | 0 | 0 | 1 |
| Clenbuterol | 1 | 1 | 1 | 0 | 0 | 3 |
| Tribulus Terrestris | 0 | 1 | 1 | 0 | 0 | 2 |
| Vanadyl Sulfate | 0 | 0 | 0 | 1 | 0 | 1 |

Distribution of other drugs and phytotherapeutics intake in the population expressed as absolute values. BB_INS_, bodybuilders abusing insulin alone (n=1) or in association with anabolic androgenic steroids (n=3) or with anabolic androgenic steroids and growth hormone (n=11); BB_GH_, bodybuilders abusing growth hormone alone (n=1) or in association with anabolic androgenic steroids (n=11); BB_AAS_, bodybuilders abusing only anabolic androgenic steroids; BB_NU_, bodybuilders not using hormones; CTRL, non-bodybuilders. IGF-1, Insulin growth factor -1

**Table S3. – Insulin administration**

| **Timing** | **%** |
| --- | --- |
| After training/Before breakfast | 29 |
| After training/After breakfast/Before sleep | 7 |
| Before and after training | 14 |
| Before training | 21 |
| Before training/After breakfast | 7 |
| After training/After breakfast | 14 |
| After breakfast | 7 |
|  |  |
| **drug type** | **%** |
| Rapid | 85 |
| Rapid+ultralente | 8 |
| regular | 8 |
|  |  |
| **dosage** | **%** |
| 1xweek | 7 |
| Few weeks/months per year | 53 |
| 6-8 weeks | 7 |
| 3 months | 7 |
| 1 week/month per year | 20 |
| Almost all weeks/months per year | 7 |

N=15, Details on the type and dosages of insulin used expressed as percent distribution among bodybuilders.
